# Thalassospiramide G, a New γ-Amino-Acid-Bearing Peptide from the Marine Bacterium *Thalassospira* sp

**DOI:** 10.3390/md11030611

**Published:** 2013-02-26

**Authors:** Soohyun Um, Yuna Pyee, Eun-Hee Kim, Sang Kook Lee, Jongheon Shin, Dong-Chan Oh

**Affiliations:** 1 Natural Products Research Institute, College of Pharmacy, Seoul National University, Seoul 151-742, Korea; E-Mails: ush2727@snu.ac.kr (S.U.); Yvette11@snu.ac.kr (Y.P.); sklee61@snu.ac.kr (S.K.L.); shinj@snu.ac.kr (J.S.); 2 Division of Magnetic Resonance, Korea Basic Science Institute, Ochang, Chungbuk 363-883, Korea; E-Mail: keh@kbsi.re.kr

**Keywords:** unicellular bacteria, marine bacteria, *Thalassospira*, thalassospiramide, γ-amino acid, iNOS assay

## Abstract

In the chemical investigation of marine unicellular bacteria, a new peptide, thalassospiramide G (**1**), along with thalassospiramides A and D (**2**–**3**), was discovered from a large culture of *Thalassospira* sp. The structure of thalassospiramide G, bearing γ-amino acids, such as 4-amino-5-hydroxy-penta-2-enoic acid (AHPEA), 4-amino-3,5-dihydroxy-pentanoic acid (ADPA), and unique 2-amino-1-(1*H*-indol-3-yl)ethanone (AIEN), was determined via extensive spectroscopic analysis. The absolute configuration of thalassospiramide D (**3**), including 4-amino-3-hydroxy-5-phenylpentanoic acid (AHPPA), was rigorously determined by ^1^H–^1^H coupling constant analysis and chemical derivatization. Thalassospiramides A and D (**2**–**3**) inhibited nitric oxide (NO) production in lipopolysaccharide (LPS)-stimulated mouse macrophage RAW 264.7 cells, with IC_50_ values of 16.4 and 4.8 μM, respectively.

## 1. Introduction

The discovery of new bioactive natural products from marine microorganisms has been the most rapidly expanding field in marine natural products research over the past fifteen years [1]. Ever since marine microbes were highlighted as an emerging resource for bioactive molecules by Fenical [2], they have demonstrated their pharmaceutical potential by providing structurally novel natural products for drug discovery, such as salinosporamide A, which is currently in clinical trials. Drugs derived from microorganisms could overcome the supply issues inherent in macro-organism-derived drugs [3]. To date, most investigations of marine microbial secondary metabolites have focused on organisms that produce large numbers of natural products, such as actinomycetes, fungi and cyanobacteria, leaving unicellular bacteria a relatively unexplored resource [4]. 

However, recent chemical studies of marine unicellular bacteria, primarily unicellular proteobacteria, have indicated that this group could be an abundant source of bioactive small molecules. Among the phylogenetically diverse marine proteobacteria, γ-proteobacteria exhibit the most chemically diverse bioactive natural products [5]. These molecules include the recent discoveries of cyclic tetrapeptides from *Pseudomonas* sp. associated with the seaweed *Diginea* sp. [6], the tricyclic antibiotic, zafrin, from *Pseudomonas stutzeri* found in the intestinal tract of ribbonfish [7] and the amphiphilic siderophores, loihichelins, from *Halomonas* [8]. *Pseudoalteromonas* sp. also yielded antimicrobial polybrominated secondary metabolites [9], and two distinctive *Vibrio* spp. produced the tris-catechol amide siderophore, trivanchrobactin [10] and the maleimides, aqabamycins [11,12], respectively. Although relatively few secondary metabolites have been reported from marine α-proteobacteria, causing this class to be overlooked as a source of new bioactive small molecules, pioneering studies have led to the isolation of agrochelin, a cytotoxic thiazole alkaloid from *Agrobacterium* [13] and B-90063, a dimeric oxazole pyridone analog from *Blastobacter* [14], which indicate the biomedical potential of this class of proteobacteria. In addition, an early investigation of the α-proteobacterium *Thalassospira* sp., strain number CNJ328, resulted in the discovery of unique immunosuppressive peptides, thalassospiramides A and B [15]. Further studies of the thalassospiramides and their biosynthesis recently reported more new analogues from *Tistrella* and *Thalassospira* isolates and the biosynthetic pathway [16].

**Figure 1:**
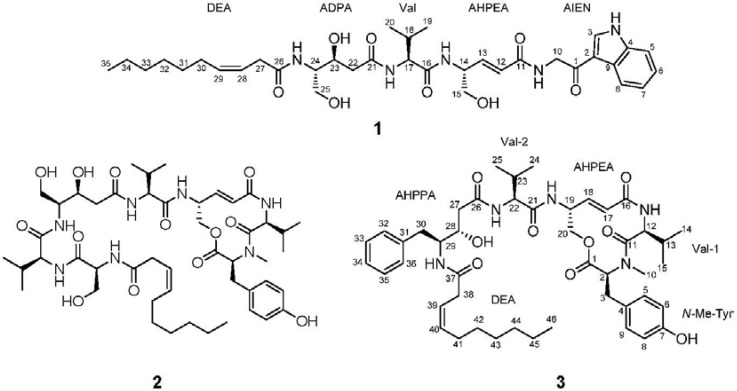
The structures of thalassospiramides G, A and D (**1**–**3**).

In our search for new bioactive natural products from marine unicellular bacteria, we cultivated a *Thalassospira* strain, CNJ328, chemically investigated its culture and discovered the production of an additional γ-amino-acid-bearing peptide, thalassospiramide G, along with thalassospiramides A and D (Figure 1). For biological evaluation, we performed a bioassay measuring the inhibition of nitric oxide (NO) production in lipopolysaccharide (LPS)-stimulated mouse macrophage. NO is considered a cellular signaling molecule and is formed by catalyzing nitric oxide synthase (NOS) from a substrate, l-arginine. Even though the production of NO by macrophages serves as a host defense against pathogens, the overproduction of NO and its corresponding enzyme, inducible nitric oxide synthase (iNOS), are highly associated with inflammation. Therefore, the regulation of NO production and iNOS expression is considered a useful target for the procurement of anti-inflammatory agents [17]. We report the structural determination of a new peptide, thalassospiramide G, the stereochemistry of thalassospiramide D and the inhibitory activities of thalassospiramides A, D and G against nitric oxide (NO) production.

## 2. Results and Discussion

### 2.1. Structural Elucidation

Thalassospiramide G (**1**) was isolated as an oil with a molecular ion at *m/z* 670.3785 [M + H]^+^. The molecular formula was determined to be C_35_H_51_N_5_O_8_ (unsaturation number: 13) based on high-resolution electrospray ionization (HR-ESI) mass spectrometry and ^1^H and ^13^C nuclear magnetic resonance (NMR) data (Table 1). Signals in the ^1^H, ^13^C and heteronuclear single quantum correlation (HSQC) NMR spectra indicated that thalassospiramide G is a peptide-derived compound with four characteristic amide NH signals (δ_H_ 8.37, 8.18, 7.92 and 7.54), three methine (δ_H_ 4.25–δ_C_ 57.5; δ_H_ 3.70–δ_C_ 54.6; δ_H_ 4.47–δ_C_ 51.7) and one methylene (δ_H_ 4.52–δ_C_ 45.5) signal, each bearing a nitrogen atom and four amide ^13^C signals (δ_C_ 171.2, 171.0, 170.6 and 164.9). Further analysis of the ^1^H and ^13^C NMR spectrum revealed that thalassospiramide G possesses unusual olefinic protons (δ_H_ 6.62, 6.13, 5.54, 5.46) and one ketone signal (δ_C_ 190.2), indicating that this is a modified peptide. The UV spectrum exhibited absorption maxima at 204, 239 and 280 nm, which differed substantially from previously-reported thalassospiramide A (**2**). Its distinct UV spectrum also indicated that thalassospiramide G bears a unique chromophore uncommon in peptide-derived compounds, whereas thalassospiramides A and D possess only aromatic rings as chromophores. Careful interpretation of the ^13^C and HSQC NMR spectra allowed for the complete assignment of the one-bond carbon–proton correlations and quaternary carbons, which included 14 methines, 10 methylenes and three methyls with eight quaternary carbons. 

Given this information, a valine could be identified in a straightforward manner using the combined analyses of the correlation spectroscopy (COSY), total correlation spectroscopy (TOCSY) and heteronuclear multiple bond correlation (HMBC) NMR spectra. In addition to this standard amino acid unit, two γ-amino acid units, a hydrophobic chain and an amino acid-derived substructure were identified. The unusual olefinic protons (H-12 and H-13) at δ_H_ 6.13 and 6.63 were located in a *trans*-double bond (*J* = 16.0 Hz) and were connected to H-14 (δ_H_ 4.47), as indicated by the COSY and TOCSY NMR spectra. H-14 displayed further COSY correlations to 14-NH (δ_H_ 8.18) and H_2_-15 (δ_H_ 3.46, 3.44), connecting 4-amino-5-hydroxy-penta-2-enoic acid (AHPEA) with the carbonyl carbon (C-11; δ_C_ 164.9), based on the HMBC correlations of H-12 and H-13 to C-11. The COSY and TOCSY correlations among H_2_-22 (δ_H_ 2.35, 2.17), H-23 (δ_H_ 4.12), H-24 (δ_H_ 3.70), H_2_-25 (δ_H_ 3.46, 3.32) and 24-NH (δ_H_ 7.54) indicated another γ-amino acid backbone. Further analysis of the COSY and HMBC correlations provided the assignment of 4-amino-3,5-dihydroxy-pentanoic acid (ADPA), including the amide carbon, C-21 (δ_C_ 171.2). The final substructure, dec-3-enoic acid (DEA), was also determined via COSY and HMBC NMR analysis. The rotating-frame nuclear Overhauser effect correlation spectroscopy (ROESY) correlation between the olefinic protons (δ_H_ 5.54, 5.46) indicated that the double bond in this hydrophobic chain possessed *cis* geometry. After assigning these amino acids and the hydrophobic chain, ten carbon (δ_C_ 190.2, 136.5, 133.6, 125.4, 122.7, 121.6, 120.9, 114.0, 112.1 and 45.5) and seven proton signals (δ_H_ 8.44, 8.16, 7.50, 7.23, 7.19 and 4.52 (2H)) were left unassigned. We carefully analyzed their correlations in the COSY, TOCSY, HSQC and HMBC spectra and deduced an indole moiety (C-2 to C-9). The COSY correlations and multiplicities of the four aromatic protons (δ_H_ 8.16 d, 7.50 dd, 7.23 d, 7.19 dd) constructed an *ortho*-substituted aromatic ring (C-4 to C-9). The last aromatic proton signal at δ_H_ 8.44 showed HMBC correlations to the quaternary carbons C-2 (δ_C_ 114.0), C-4 (δ_C_ 133.6) and C-9 (δ_C_ 125.4). Along with these correlations, the three-bond heteronuclear couplings from H-5 (δ_H_ 7.50) to C-9 and from H-8 (δ_H_ 8.16) to C-4 confirmed the indole moiety. An additional COSY correlation between an amide NH (10-NH; δ_H_ 8.37) and H_2_-10 (δ_H_ 4.52) assigned the connectivity of the amide proton (10-NH) to the methylene (H_2_-10). The indole and methylene (H_2_-10) were connected through the ketone carbon at δ_C_ 190.2 by the ROESY couplings between H_2_-10 (δ_H_ 4.52) and H-3 (δ_H_ 8.44), yielding a partial structure of 2-amino-1-(1*H*-indol-3yl)ethanone (AIEN), which serves as an additional unique residue in **1**. 

**Table 1:** Nuclear magnetic resonance (NMR) Data for **1** in dimethyl sulfoxide (DMSO)-*d*_6_.

C/H	δ_H_^ a^	mult (*J* in Hz)	δ_C_^ b^	
1			190.2	C
2			114.0	C
3	8.44	s	133.6	CH
4			136.5	C
5	7.50	d (8.0)	112.1	CH
6	7.23	dd (7.5, 7.0)	122.7	CH
7	7.19	dd (7.5, 7.0)	121.6	CH
8	8.16	d (8.0)	120.9	CH
9			125.4	C
10	4.52	m	45.5	CH_2_
10-NH	8.37	t (5.5)		
11			164.9	C
12	6.13	d (16.0)	124.3	CH
13	6.62	dd (16.0, 6.0)	140.7	CH
14	4.47	m	51.7	CH
14-NH	8.18	d (8.5)		
15a	3.46	m	62.8	CH_2_
15b	3.44	m		
16			171.0	C
17	4.25	dd (9.0, 6.0)	57.5	CH
17-NH	7.92	Br, s		
18	2.05	m	30.2	CH
19	0.86	m	19.1	CH_3_
20	0.84	m	17.6	CH_3_
21			171.2	C
22a	2.35	dd (15.0, 10.0)	39.9	CH_2_
22b	2.17	dd (15.0, 4.0)		
23	4.12	m	65.9	CH
24	3.70	m	54.6	CH
24-NH	7.54	d (7.0)		
25a	3.46	m	60.0	CH_2_
25b	3.32	m		
26			170.6	C
27	2.97	m	33.9	CH_2_
28	5.54	m	123.7	CH
29	5.46	m	131.4	CH
30	2.02	m	26.5	CH_2_
31	1.31	m	28.6	CH_2_
32	1.25	m	28.2	CH_2_
33	1.23	m	30.9	CH_2_
34	1.26	m	21.9	CH_2_
35	0.86	t (6.5)	13.6	CH_3_

^a^ 900 MHz; ^b^ 225 MHz.

**Figure 2:**
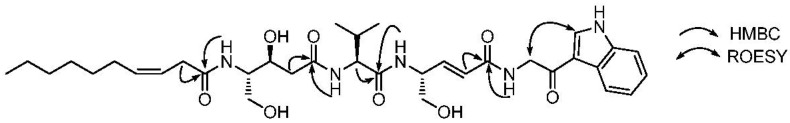
Key heteronuclear multiple bond correlation (HMBC) and rotating-frame nuclear Overhauser effect correlation spectroscopy (ROESY) correlations in thalassospiramide G.

The five fragments (valine, AHPEA, ADPA, DEA and AIEN) were then connected by careful analysis of the HMBC correlations. The long-range correlations from 10-NH to C-11 (δ_C_ 164.9) and the ROESY correlation between 10-NH and H-12 (δ_H_ 6.13) confirmed the connectivity of AIEN to AHPEA. The amide proton (δ_H_ 8.18) of AHPEA indicated an HMBC correlation to C-16, the carbonyl carbon of valine, connecting AHPEA and valine. The HMBC correlations of the NH of valine and H_2_-22 in ADPA to the amide carbon (C-21) of AHPPA established the sequence of valine to ADPA. The DEA was located next to ADPA based on the heteronuclear coupling between 25-NH and the carbonyl carbon of DEA, completing the planar structure of thalassospiramide G (**1**) (Figure 2). 

The relative configuration of the ADPA unit was established via ^1^H–^1^H coupling constant analysis based on the method for determining the relative stereochemistry of statine units [18]. The large vicinal coupling (10.0 Hz) of the downfield proton (δ_H_ 2.35) of H_2_-22 clearly indicated an anti-configuration between the substituents on C-23 and C-24. The yield of thalassospiramide G (**1**) was too low (0.9 mg/160 L culture) for chemical derivatization to determine the absolute configuration. The three consecutive units bearing stereogenic centers (AHPEA, Val and ADPA) were also found in thalassospiramide A (**2**), which was isolated from the same strain. Therefore, the absolute configurations of the three units in **1** were assumed to be identical to those in **2**, because the identical moieties in **1** and **2** were most probably produced through the same biosynthetic modules in strain CNJ328.

Along with thalassospiramide G, two more thalassospiramide analogues were purified. The major compound was identified as thalassospiramide A (**2**) based on the original report by a part of the authors [15]. The last compound (**3**) was purified as a gum, with the molecular formula C_46_H_65_N_5_O_9_, as determined from the molecular ion at *m/z* 832.4825 [M + H]^+^ (832.4855 calculated for C_46_H_66_N_5_O_9_) via high-resolution ESI mass spectrometry and ^1^H and ^13^C NMR spectroscopy. The molecular formula is identical to one of the analogues very recently reported [16]. Careful comparison of the NMR data and molecular formula of **3** with the literature values [16] enabled us to identify the compound as thalassospiramide D and revise the incorrect NMR assignments in 4-amino-3-hydroxy-5-phenylpentanoic acid (AHPPA) (Table S1). We also provided physiochemical data for thalassospiramide D that was missing in the literature (see Experimental Section).

The configuration of the 4-amino-3-hydroxy-5-phenylpentanoic acid (AHPPA) unit in the family of the thalassospiramides previously has never been rigorously determined. Only the relative configuration of AHPPA in thalassospiramide B was proposed by analysis of ^1^H coupling constants and ROESY correlations [15]. Recent development of the method for relative configurations of statine units used for **1** [18] enabled confident establishment of the relative configuration of AHPPA. The down-field proton signal (δ_H_ 3.08) of the H_2_-27 methylene pair displayed a large vicinal coupling (8.0 Hz) to the H-29 (δ_H_ 4.65), which indicates an anti-configuration between the hydroxy group at C-28 and the side-chain alkyl group at C-29. To determine the absolute configuration of the AHPPA, thalassospiramide D (**3**) was subjected to flash acid hydrolysis for 1 h [19], and the free amino acids were derivatized using l- and d-Marfey’s reagents (l- and d-1-fluoro-2,4-dinitrophenyl-5-leucine amide, FDLA). The derivatives were analyzed via LC/MS to confirm the absolute configurations of *N*-methyltyrosine and the two valines as l. The absolute configuration of the C-29 in AHPPA was deduced using the previously reported Marfey derivatization and the analyses of the four diastereomers of AHPPA [20]. According to the literature, the l-Marfey product of 4*S*-AHPPA elutes faster than its d-Marfey derivative, regardless of the absolute configuration of the C-3 in AHPPA. This result indicates that the phenyl side chain of the molecule is more hydrophobic than the β-hydroxy acid portion. This elution order was also supported by a comparison of the retention times of the l-FDLA derivatives of 2-phenylethanamine and 4-amino-3-hydroxybutanoic acid, the fragments of AHPPA (see Experimental Section). We observed faster elution of the l-Marfey product for the AHPPA than the d-Marfey derivative, confirming the absolute configuration of C-29 as *S* and, subsequently, assigning the 28*S* configuration. 

Thalassospiramide G (**1**) is structurally unique and the most distinct member of the thalassospiramide family, because it incorporates 2-amino-1-(1*H*-indol-3yl)ethanone (AIEN). To the best of our knowledge, AIEN has been reported only once in a natural product, a putative biogenic peptidic precursor of the indole alkaloid, almazole C, isolated from a Senegalese delesseriacean seaweed [21]. Our report of thalassospiramide G is the first to describe an AIEN unit in a natural product from an organism other than seaweed. The recent discovery of the biosynthetic gene cluster of the thalassospiramide family elucidated the formation of the repeating γ-amino acid unit. By iterative employment of the specific modules, the bacterium synthesizes the common valine-γ-amino acid substructures multiple times. Then, it utilizes the final two modules in the cluster and couples the peptide chain with an additional valine and *N*-methyltyrosine, which cyclizes the 12-membered lactone ring in the thalassospiramides. However, the biosynthesis of thalassospiramide G cannot be fully understood based on the reported biosynthetic modules, opening up the possibility of the existence of a separate module elongating the peptide chain by adding tryptophan, which later possibly undergoes decarboxylation and oxidation at β-carbon.

### 2.2. Inhibition of NO Production in LPS-Stimulated RAW 264.7 Cells

**Figure 3:**
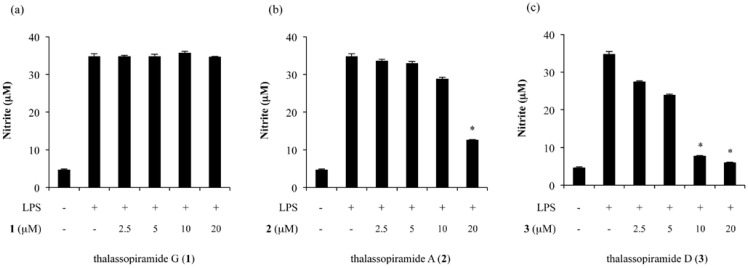
Effects of (**a**) thalassospiramide G (**1**), (**b**) thalassospiramide A (**2**) and (**c**) thalassospiramide D (**3**) on nitric oxide production.

To determine the biological activity of the thalassospiramides, we tested their effects on the production of NO induced by lipopolysaccharide (LPS). To evaluate the inhibitory effects of thalassospiramides G, A and D (**1**–**3**) on the production of NO, RAW 264.7 cells were treated with LPS, and the quantity of nitrite, a stable metabolite of NO, was measured in the media. As shown in Figure 3, the treatment with LPS markedly increased the production of NO from a basal level of 4.7 ± 0.2 μM to 34.8 ± 0.7 μM after 20 h of incubation. In this assay, l-NMMA (l-NG-monomethylarginine, 50 μM, a non-selective inhibitor of NOS) was used as a positive control and reduced the NO content by 70.9% (data not shown). No significant effect on NO production was observed for thalassospiramide G (Figure 3a). However, when the cells were pretreated with varying concentrations of thalassospiramides A (**2**) and D (**3**) (0–20 μM for 30 min prior to LPS stimulation), the NO production was significantly inhibited in a concentration-dependent manner (Figure 3b,c). Thalassospiramides A (**2**) and D (**3**) exhibited significant inhibitory activity on the NO production, with IC_50_ values of 16.4 μM and 4.8 μM, respectively, without observable cytotoxicity up to 20 μM, as determined using an MTT assay (supplementary data, Figure S17). 

## 3. Experimental Section

### 3.1. General Experimental Procedures

Optical rotations were acquired on a Jasco P-1020 polarimeter with a 5-cm cell. The UV spectra were recorded using a Perkin Elmer Lambda 35 UV-Vis spectrophotometer with a 1-cm cell. The IR spectra were obtained on a JASCO FT-IR-4200 spectrometer. The low-resolution LC/MS data were acquired with an Agilent Technologies 6130 quadrupole mass spectrometer in tandem with an Agilent Technologies 1200 series HPLC using a reversed-phase column (Phenomenex Luna C_18_ (2), 4.6 mm × 100 mm). The high-resolution ESI mass spectra were acquired with a Thermo Scientific LTQ-Orbitrap mass spectrometer. The NMR spectra were collected on a Bruker 900 MHz NMR spectrometer at the Korea Basic Science Institute in Ochang.

### 3.2. Bacterial Material, Cultivation and Extraction

The marine bacterial strain CNJ328 (GenBank accession number AY989809) was originally isolated from the surface of *Rosenvingea* sp. brown alga in the Bahamas in 1996. As described in the previous study, the 16S rDNA sequence indicated that it is most closely related to *Thalassospira lucentensis* (98% identity), indicating that this α-proteobacterium is a member of the genus *Thalassospira* [15]. The bacterium was cultivated in 40 2.8-L Fernbach flasks, each containing 1 L YPM medium (2 g yeast extract, 2 g peptone and 4 g mannitol/1 L seawater). The culture was incubated for 2 days at 27 °C with shaking at 215 rpm. For large-scale fermentation, this procedure was repeated four times to cultivate 160 L. For the solid-phase extraction, Amberlite XAD-7 resin was added to the culture (20 g/L), and the culture was shaken with the resin at 215 rpm for 2 h. The resin was retrieved by filtering the culture through cheesecloth, then washing with DI water. The organic material was extracted by shaking the resin in acetone at 215 rpm for 1 h. The acetone extract was concentrated using a rotary evaporator to yield 35 g dry, crude material from 160 L of bacterial culture.

### 3.3. Isolation of Thalassospiramides G, A and D

One quarter of the crude extract (~9 g) was fractionated through a Si vacuum column (100 g Si gel) by eluting with a step gradient of isooctane, ethyl acetate and methanol (isooctane/ethyl acetate 1:1, ethyl acetate, ethyl acetate/methanol 9:1, ethyl acetate/methanol 5:1, ethyl acetate/methanol 2:1 and methanol). The 5:1 ethyl acetate/methanol fraction was fractionated again with a semi-preparative reversed-phase HPLC (Phenomenex Luna, 5-μm C_18_ column, 10 mm × 250 mm, 2-mL/min flow rate, 280-nm UV detection) using isocratic 60% aqueous methanol. The purified thalassospiramides G and D (**1** and **3**) eluted at 35 and 47 min, respectively. The entire process was repeated four times to yield 0.9 mg of **1** and 2.5 mg of **3**. Thalassospiramide A (**2**) was isolated through the process described in the previous report [15].

#### 3.3.1. Thalassospiramide G (**1**)


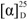
−1.5 (*c* 0.10, CH_3_CN); IR (neat) ν_max_ 3420, 1679, 1591 cm^−1^; ^1^H and ^13^C NMR data (see Table 1); UV (CH_3_CN) λ_max_(log ε) 204 (4.09), 239 (3.38), 280 (3.08); HR-ESIMS *m/z* 670.3785 [M + H]^+^ (calculated for C_35_H_52_N_5_O_8_ 670.3810).

#### 3.3.2. Thalassospiramide D (**3**)


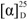
−40.5 (*c* 0.10, CH_3_CN); IR (neat) ν_max_ 3395, 2925, 1801, 1662, 1599 cm^−1^; ^1^H and ^13^C NMR data (see Table S1); UV (CH_3_CN) λ_max_(log ε) 224 (4.19), 278 (3.26); HR-ESIMS *m/z* 832.4825 [M + H]^+^ (calculated for C_46_H_66_N_5_O_9_ 832.4855).

### 3.4. Acid Hydrolysis and Advanced Marfey Analysis

A 1-mg sample of thalassospiramide D (**3**) was hydrolyzed in 0.5 mL 6 N HCl at 115 °C for 1 h. The reaction vial was then rapidly cooled in ice water for 3 min. The HCl was evaporated *in vacuo*, after which the residual HCl was removed by adding 0.5 mL of water and evaporating the solvent 3 times. The hydrolysate was completely dried under high vacuum for 12 h. The hydrolysate with free amino acids was then evenly divided into two 8-mL vials. The hydrolysate was dissolved in 100 μL of 1 N NaHCO_3_. Then, 50 μL of 10 mg/mL l-FDLA (1-fluoro-2,4-dinitrophenyl-5-l-leucine amide) in acetone was added to one of the two vials containing the dissolved free amino acids, and d-FDLA was added into the other. The reaction mixtures were incubated at 80 °C for 3 min. The mixtures were then neutralized with 50 μL of 2 N HCl, and 300 μL of a 50% solution of aqueous CH_3_CN was added. An aliquot of 10 μL of each reaction mixture was analyzed by LC/MS with a gradient solvent system (20%*–*70% CH_3_CN with 0.1% formic acid over 50 min, 0.7 mL/min flow rate, 340 nm UV detection). The *N*-methyltyrosine was eluted at retention times of 16.1 and 16.7 min with the l-FDLA and d-FDLA derivatives, respectively. The l-FDLA and d-FDLA derivatives of valine were eluted at 26.3 and 33.5 min, respectively. The l- and d-Marfey products for AHPPA were detected at 27.4 and 34.2 min, respectively. The l-FDLA derivative of 2-phenylethanamine were eluted (T_R_: 43.1 min) after that of 4-amino-3-hydroxybutanoic acid (T_R_: 21.4 min) in the LC/MS analysis.

### 3.5. Bioassay

Dulbecco’s modified Eagle’s medium (DMEM), fetal bovine serum (FBS), sodium pyruvate, l-glutamine and antibiotic-antimycotic solutions were purchased from Invitrogen™ (Grand Island, NY, USA). The lipopolysaccharide (LPS, *E. coli* 0111: B4), 3-(4,5-dimethylthiazol-2-yl)-2,5-diphenyltetrazolium bromide (MTT) and other chemicals were obtained from Sigma (St. Louis, MO, USA). The mouse macrophage RAW 264.7 cells, obtained from the American Type Culture Collection (ATCC, Rockville, MD, USA), were cultured in DMEM supplemented with 10% heat-inactivated FBS, 100 units/mL penicillin, 100 μg/mL streptomycin and 0.25 μg/mL amphotericin B. The cells were incubated at 37 °C and under 5% CO_2_ in a humidified atmosphere.

To evaluate the inhibitory activity of the test material on LPS-induced NO production, the RAW 264.7 cells in 10% FBS-DMEM without phenol red were plated in 24-well plates (3 × 10^5^ cells/mL) and incubated for 24 h. The cells were washed with PBS, taken up in fresh media and incubated with 1 μg/mL LPS in the presence or absence of the test compounds. After an additional 20-h incubation, the media were collected and analyzed for nitrite accumulation using the Griess reaction to indicate NO production—180 μL Griess reagents (0.1% *N*-(1-naphthyl)ethylenediamine dihydrochloride in H_2_O and 1% sulfanilamide in 5% H_3_PO_4_) were added to 100 μL of each supernatant from the LPS- or sample-treated cells in 96-well plates. The absorbance was measured at 540 nm, and the nitrite concentration was determined by comparison with a sodium nitrite standard curve. The percent inhibition was expressed as [1 − (NO level of test samples/NO levels of vehicle-treated control)] × 100. The IC_50_ value, the sample concentration resulting in 50% inhibition of NO production, was determined using non-linear regression analysis (% inhibition *versus* concentration). 

After completion of the Griess reaction, the MTT solution (final concentration: 500 μg/mL) was added to each well, and the cells were incubated for an additional 4 h at 37 °C. The media were discarded and dimethyl sulfoxide (DMSO) was added to each well to dissolve the generated formazan. The absorbance was measured at 570 nm, and percent survival was determined by comparison with the control group.

## 4. Conclusion

The chemical investigation of the marine unicellular bacterium *Thalassospira* sp. led to the discovery of a new peptide, thalassospiramide G (**1**), along with thalassospiramides A and D (2**–**3). The peptides are structurally unique, with unusual γ-amino acids, such as 4-amino-5-hydroxy-penta-2-enoic acid (AHPEA) and 4-amino-3,5-dihydroxy-pentanoic acid (ADPA). In addition, thalassospiramide G bears a 2-amino-1-(1*H*-indol-3-yl)ethanone (AIEN) moiety, which is quite rare in a natural product. In the LPS-induced NO production assay, thalassospiramide D displayed more significant inhibition of NO production than thalassospiramide A, indicating its potential as an anti-inflammatory agent. The structural novelty and biological activity of the secondary metabolites isolated from this marine α-proteobacterial taxonomic group suggest that marine unicellular bacteria, particularly α-proteobacteria, which have been overlooked in the search for new bioactive compounds, could potentially provide a rich source of chemically and pharmaceutically interesting natural products.

## Supplementary Files

Supplementary File 1 Supplementary Information (PDF, 768 KB)
