# Supplementary material for: Thalassospiramide G, a New γ-Amino-Acid-Bearing Peptide from the Marine Bacterium Thalassospira sp
Source: Mar Drugs. 2013 Feb 26;11(3):611–22. doi: 10.3390/md11030611 (PMC3705361; doi:10.3390/md11030611)

Supplementary Information

Figure S1. ^{1}H NMR spectrum (900 MHz) of thalassospiramide G (**1**) in DMSO-d_6. 2

Figure S2. ^{13}C NMR spectrum (225 MHz) of thalassospiramide G (**1**) in DMSO-d_6. 3

Figure S3. DEPT spectrum (225 MHz) of thalassospiramide G (**1**) in DMSO-d_6. 4

Figure S4. COSY spectrum (900 MHz) of thalassospiramide G (**1**) in DMSO-d_6. 5

Figure S5. HSQC spectrum (900 MHz) of thalassospiramide G (**1**) in DMSO-d_6. 6

Figure S6. HMBC spectrum (900 MHz) of thalassospiramide G (**1**) in DMSO-d_6. 7

Figure S7. TOCSY spectrum (900 MHz) of thalassospiramide G (**1**) in DMSO-d_6. 8

Figure S8. ROESY spectrum (900 MHz) of thalassospiramide G (**1**) in DMSO-d_6. 9

Figure S9. ^{1}H NMR spectrum (900 MHz) of thalassospiramide D (**3**) in pyridine-d_5. 10

Figure S10. ^{13}C NMR spectrum (225 MHz) of thalassospiramide D (**3**) in pyridine-d_5. 11

Figure S11. DEPT spectrum (225 MHz) of thalassospiramide D (**3**) in pyridine-d_5. 12

Figure S12. COSY spectrum (900 MHz) of thalassospiramide D (**3**) in pyridine-d_5. 13

Figure S13. HMQC spectrum (900 MHz) of thalassospiramide D (**3**) in pyridine-d_5. 14

Figure S14. HMBC spectrum (900 MHz) of thalassospiramide D (**3**) in pyridine-d_5. 15

Figure S15. TOCSY spectrum (900 MHz) of thalassospiramide D (**3**) in pyridine-d_5. 16

Figure S16. ROESY spectrum (900 MHz) of thalassospiramide D (**3**) in pyridine-d_5. 17

Table S1. NMR Data for **3** in pyridine-d_5. 18

Figure S17. Effects of thalassospiramides G, A and D on LPS-induced cell cytotoxicity. 19

Figure S1. ^{1}H NMR spectrum (900 MHz) of thalassospiramide G (**1**) in DMSO-d_6.

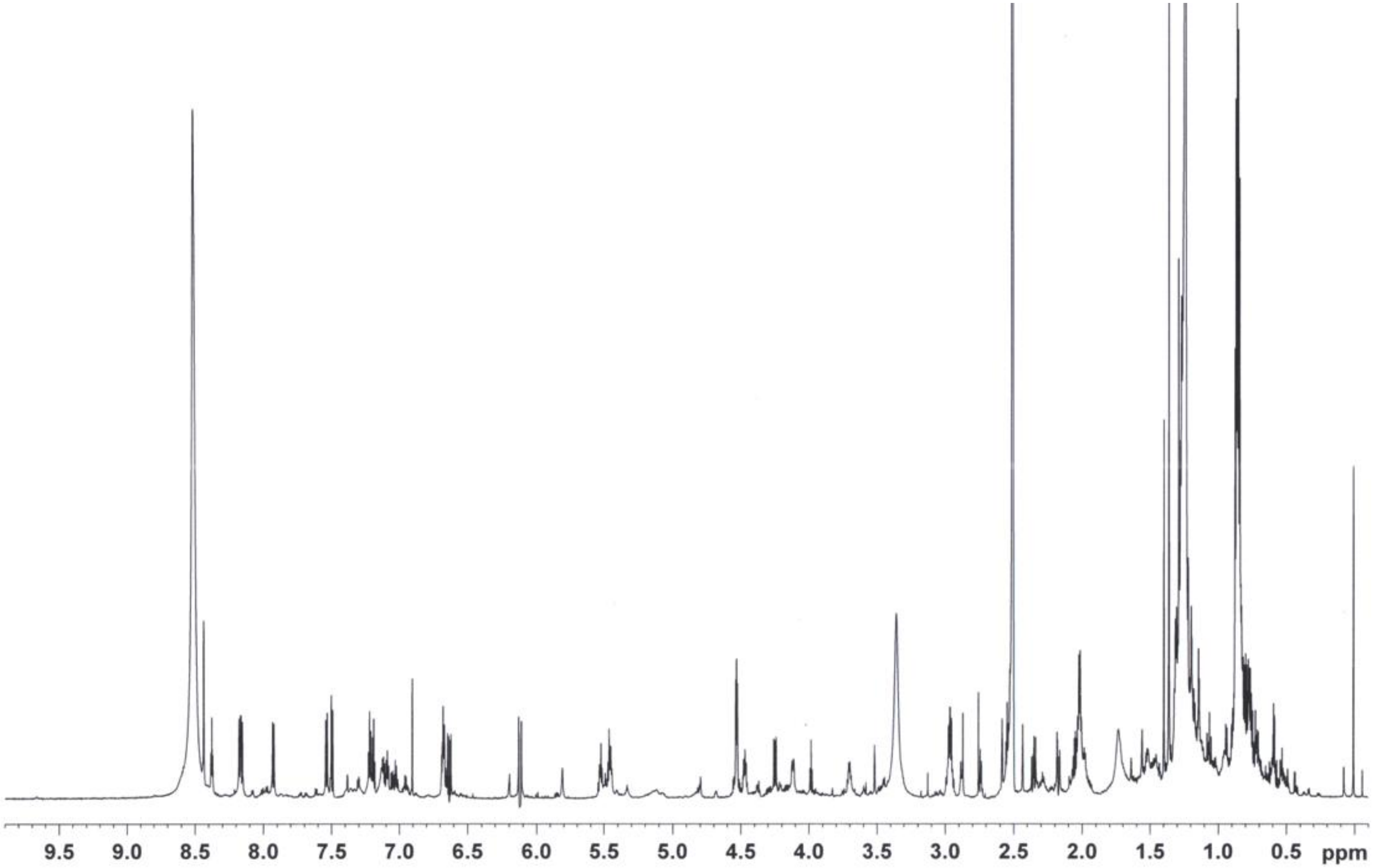

Figure S2. ^{13}C NMR spectrum (225 MHz) of thalassospiramide G (**1**) in DMSO-d_6.

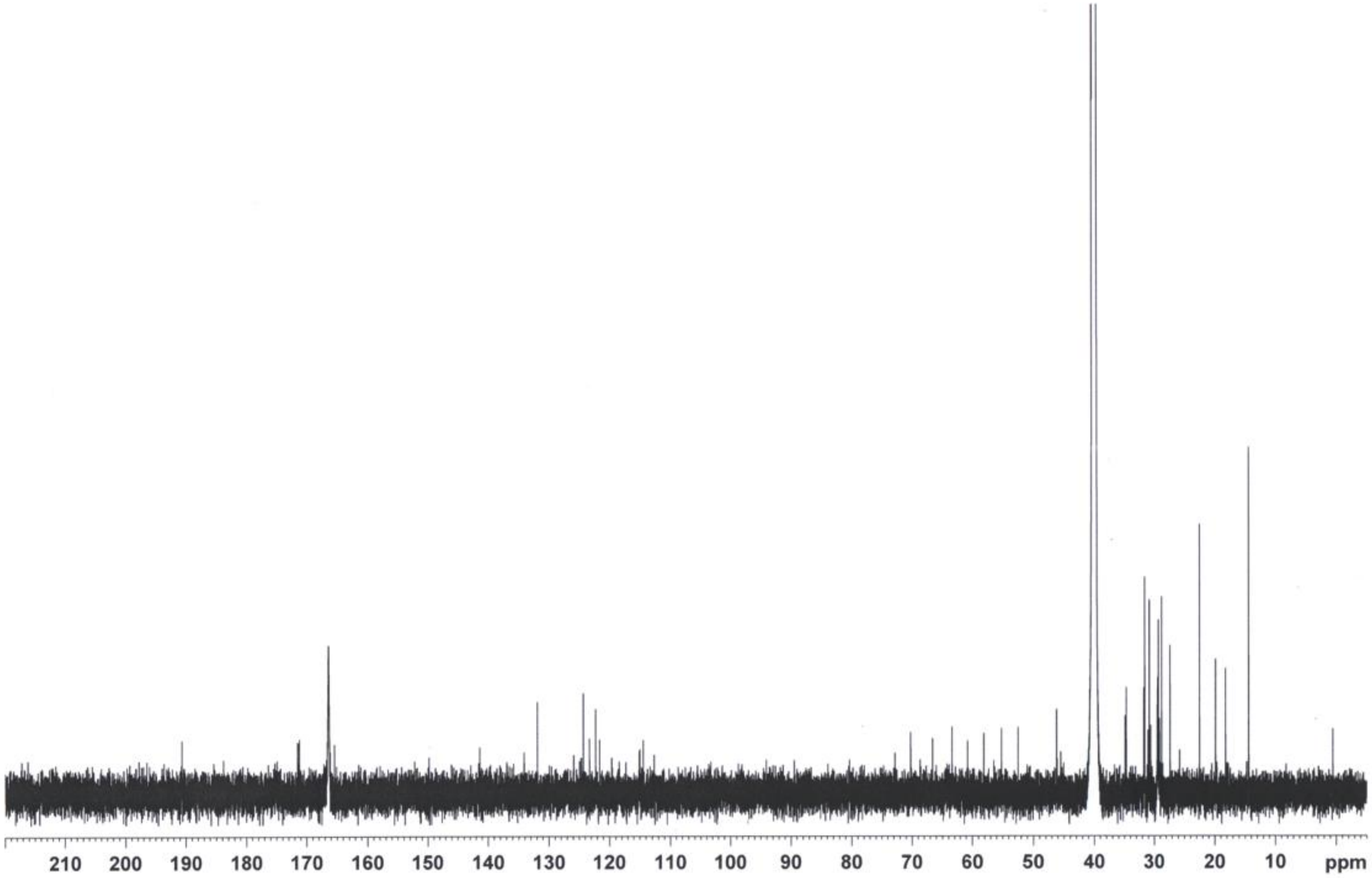

Figure S3. DEPT spectrum (225 MHz) of thalassospiramide G (**1**) in DMSO-d_6.

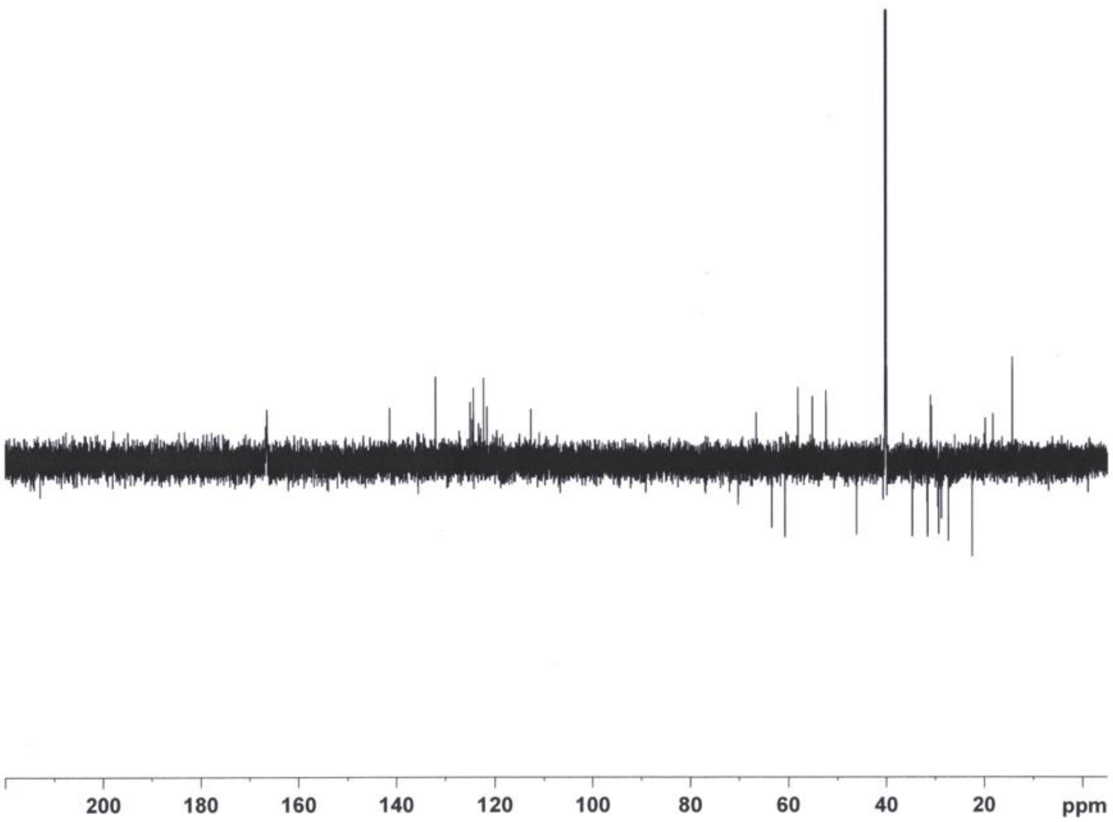

Figure S4. COSY spectrum (900 MHz) of thalassospiramide G (**1**) in DMSO-d_6.

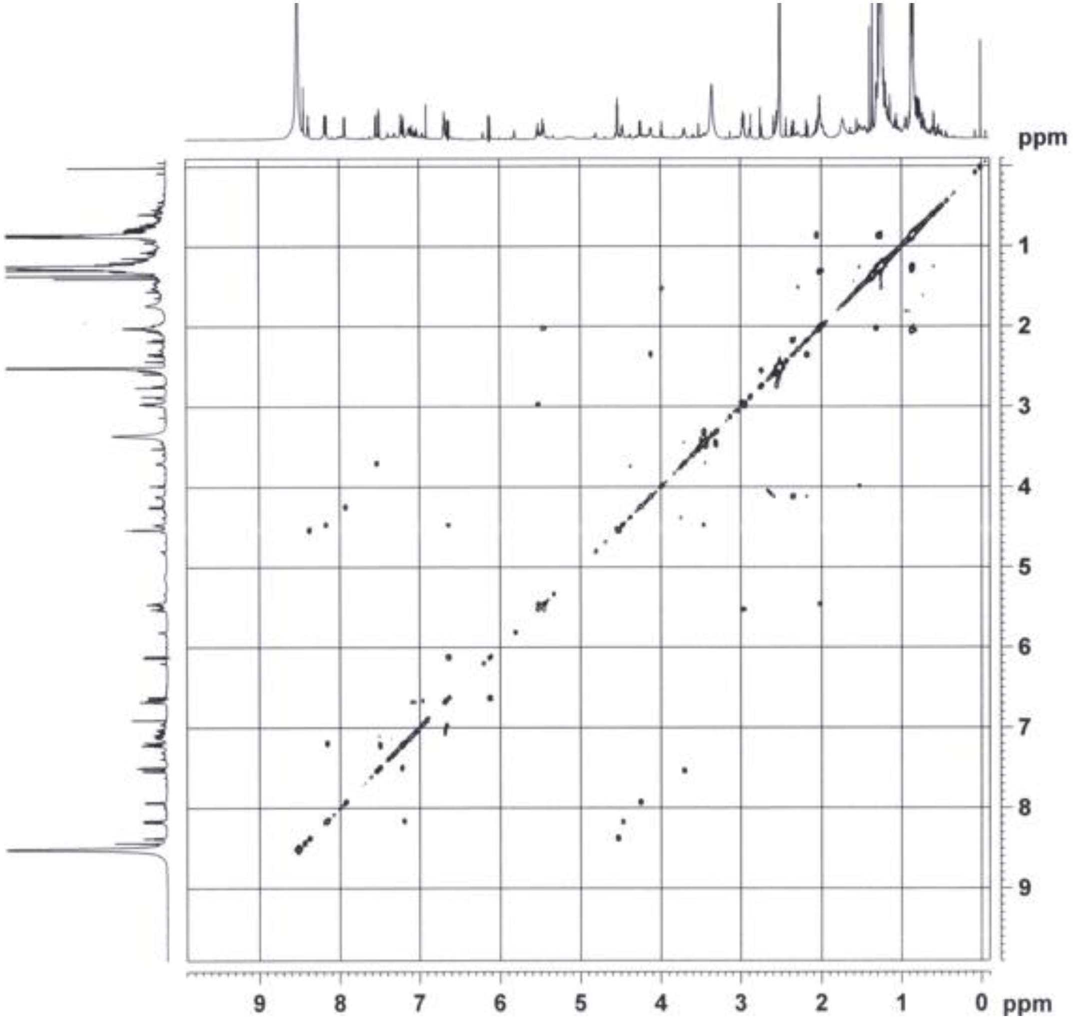

Figure S5. HSQC spectrum (900 MHz) of thalassospiramide G (**1**) in DMSO-d_6.

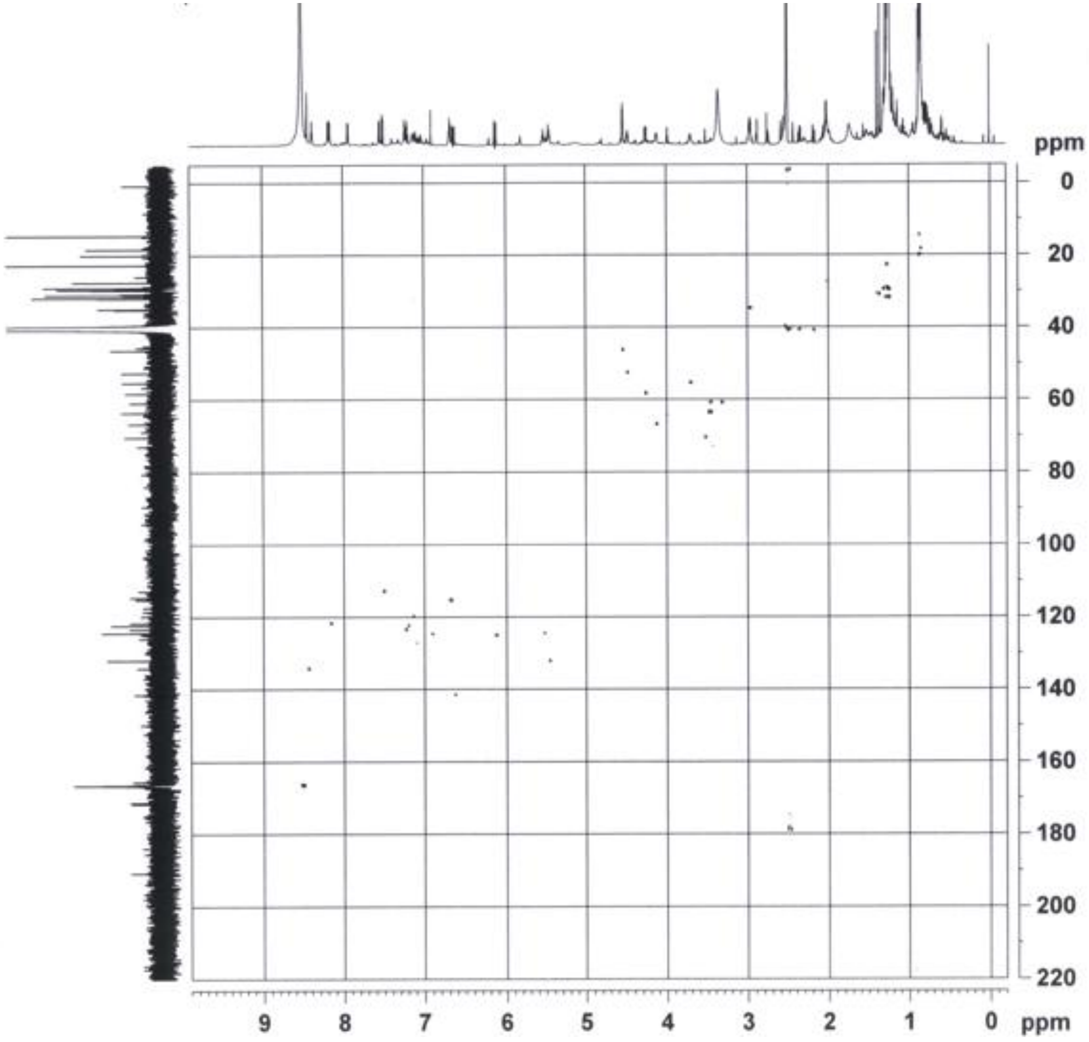

Figure S6. HMBC spectrum (900 MHz) of thalassospiramide G (**1**) in DMSO-d_6.

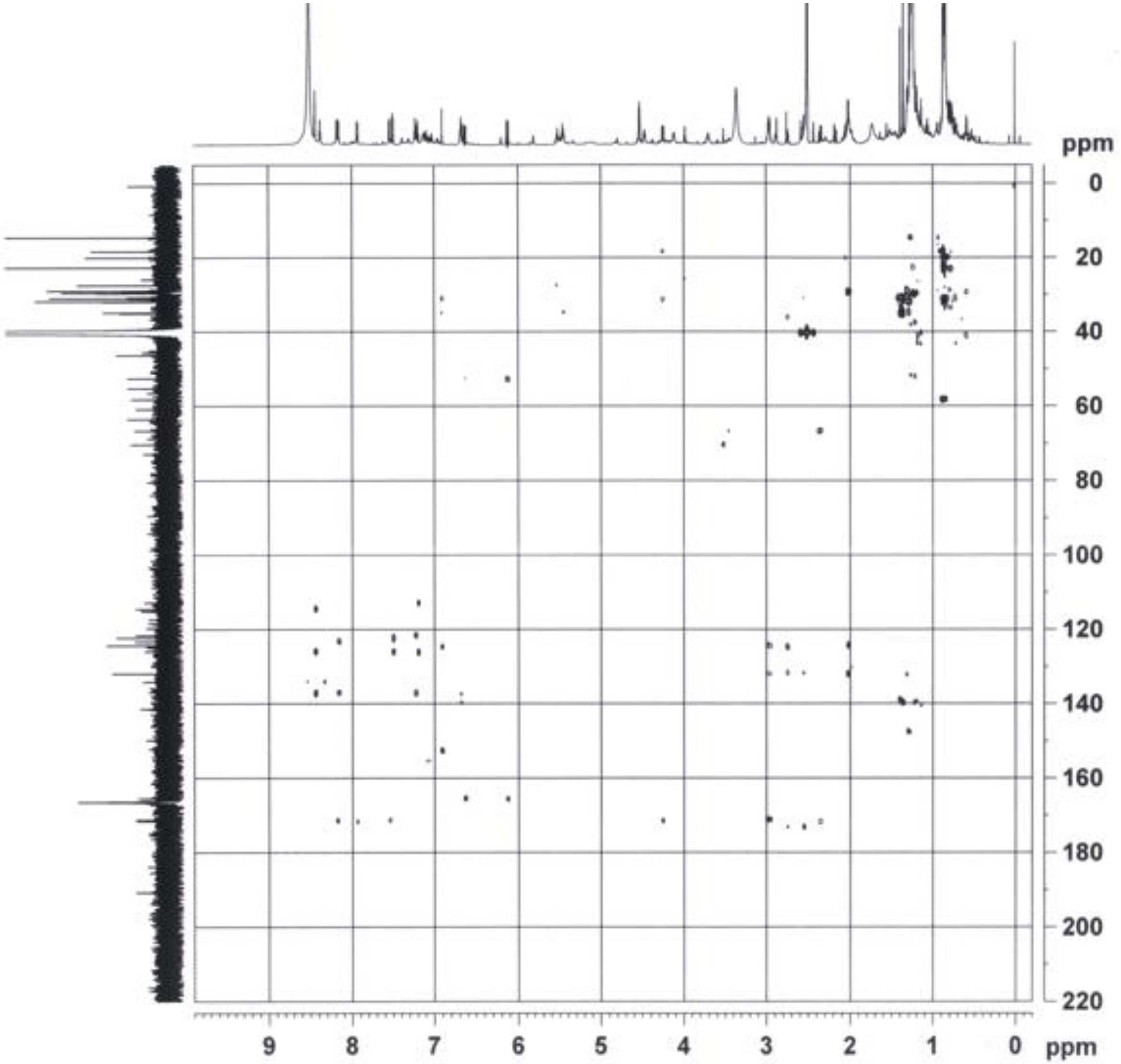

Figure S7. TOCSY spectrum (900 MHz) of thalassospiramide G (**1**) in DMSO-d_6.

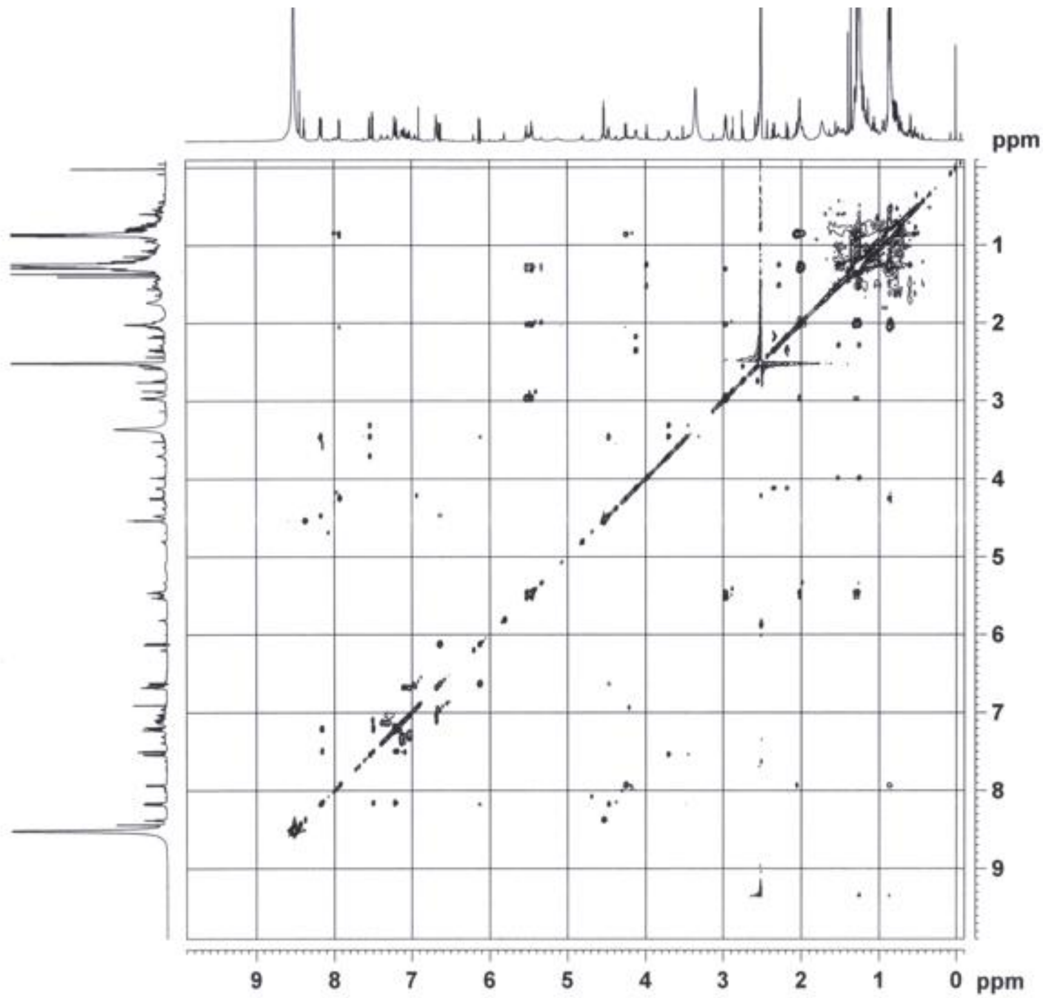

Figure S8. ROESY spectrum (900 MHz) of thalassospiramide G (**1**) in DMSO-d_6.

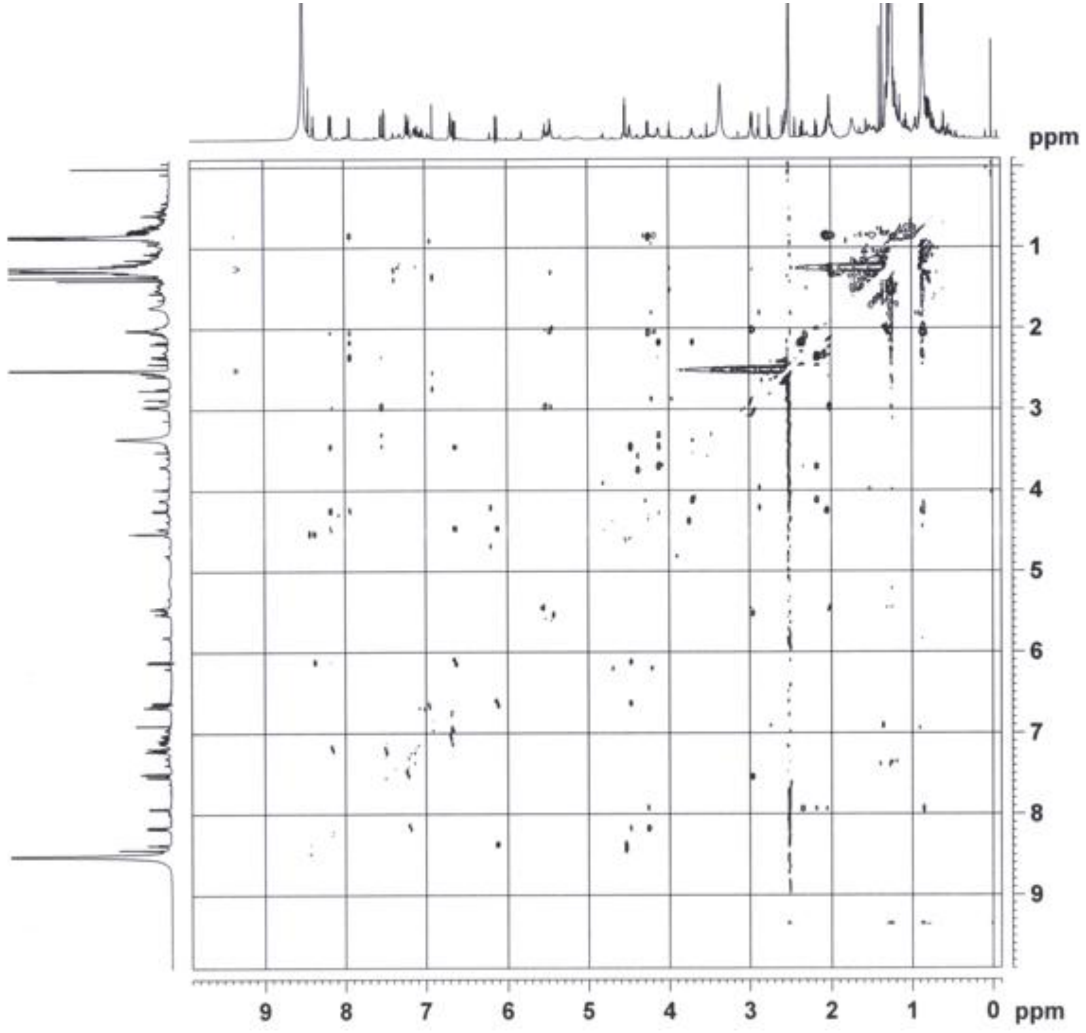

Figure S9. ^{1}H NMR spectrum (900 MHz) of thalassospiramide D (**3**) in pyridine-d_5.

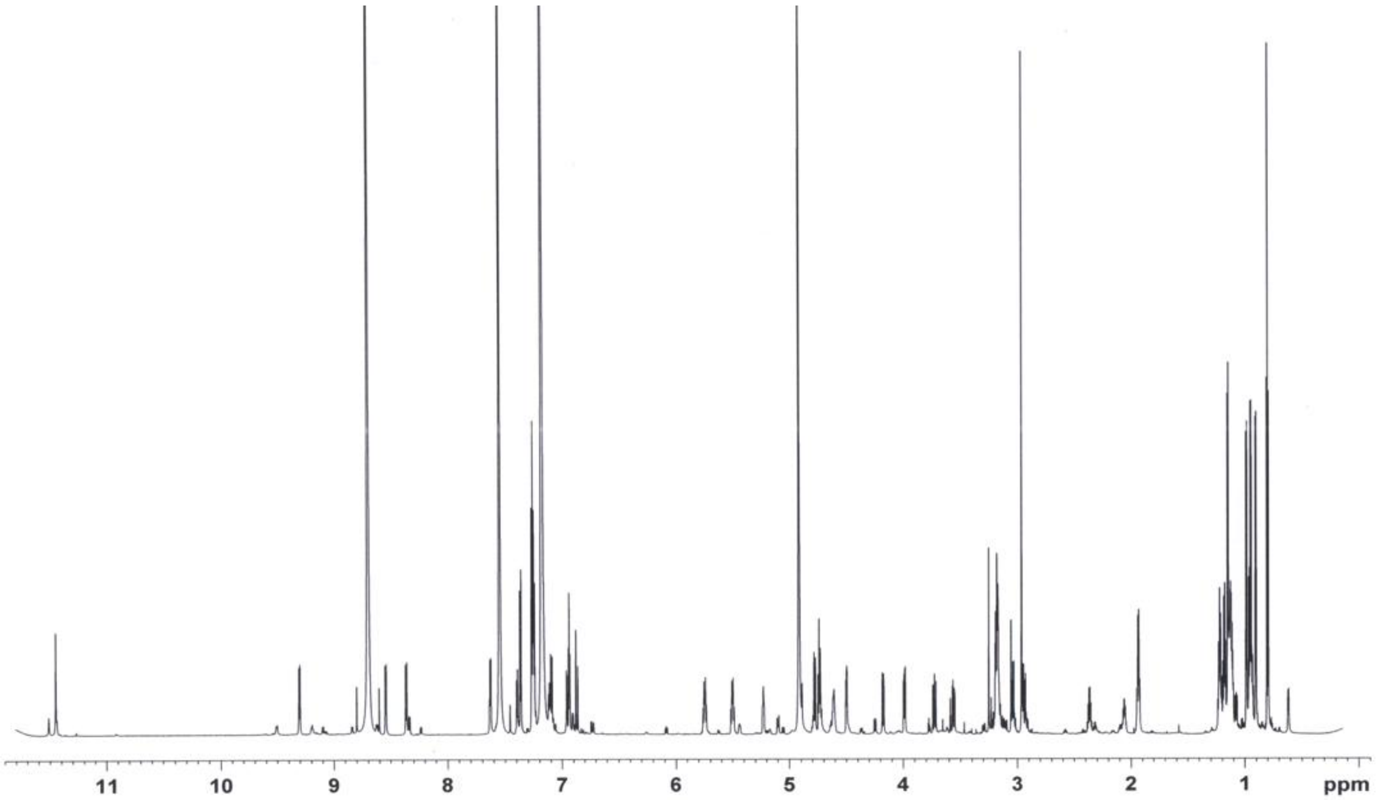

Figure S10. ^{13}C NMR spectrum (225 MHz) of thalassospiramide D (**3**) in pyridine-d_5.

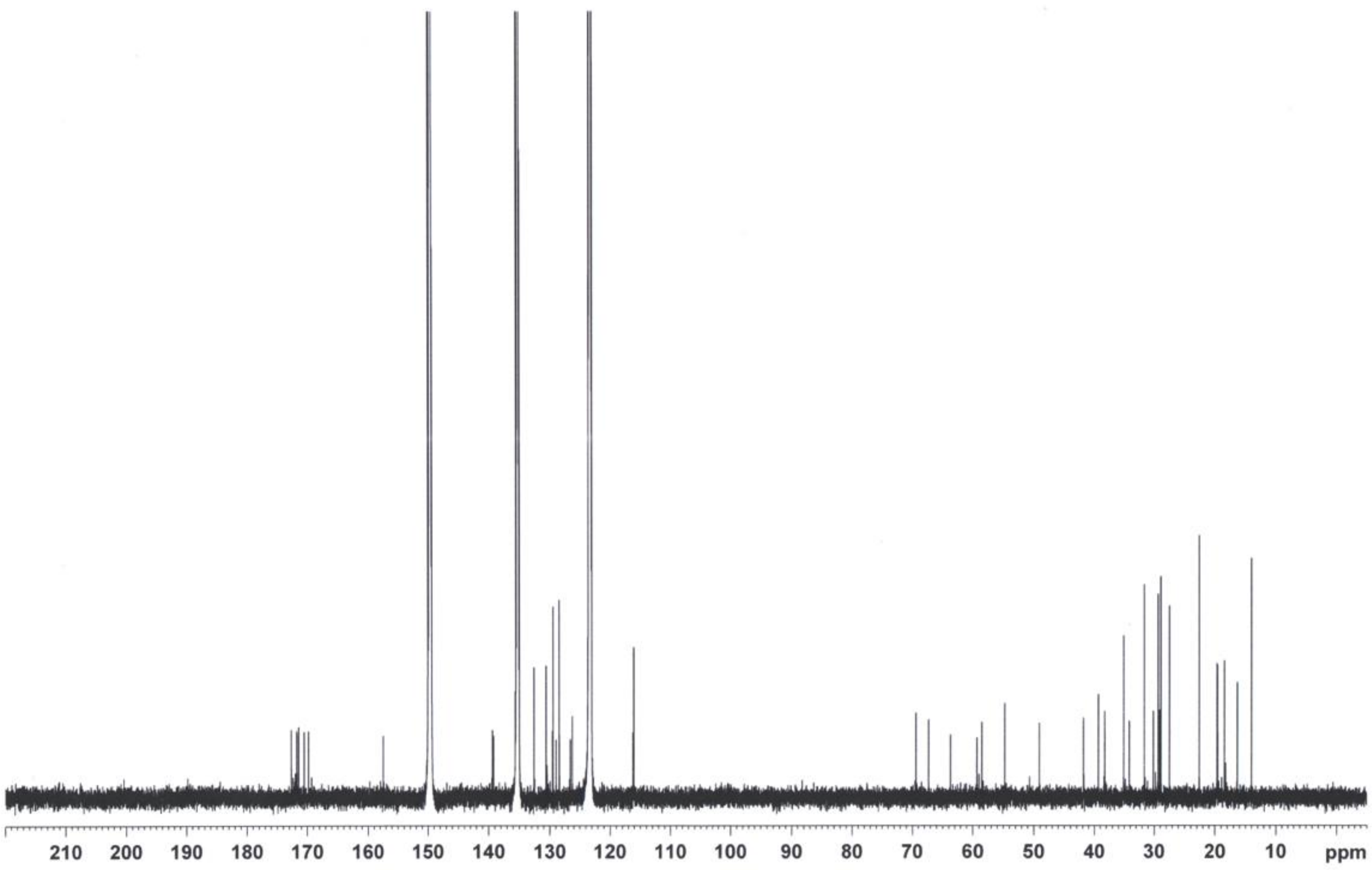

Figure S11. DEPT spectrum (225 MHz) of thalassospiramide D (**3**) in pyridine-d_5.

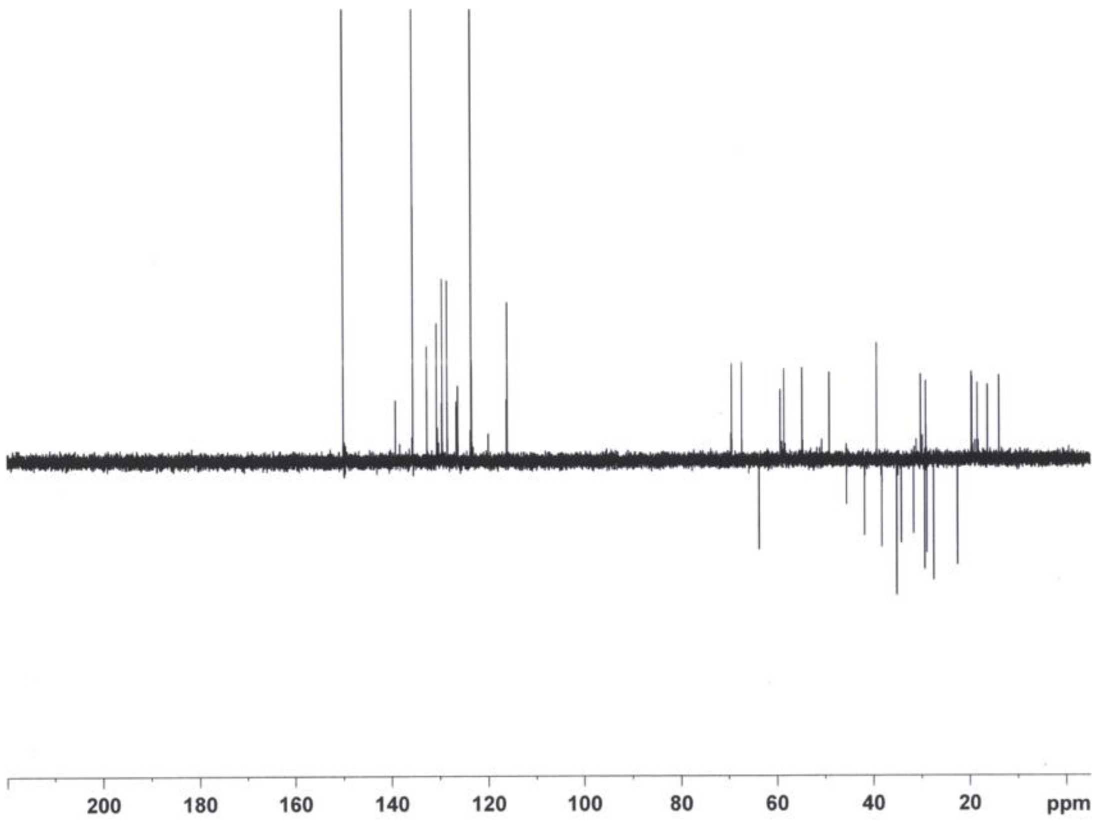

Figure S12. COSY spectrum (900 MHz) of thalassospiramide D (**3**) in pyridine-d_5.

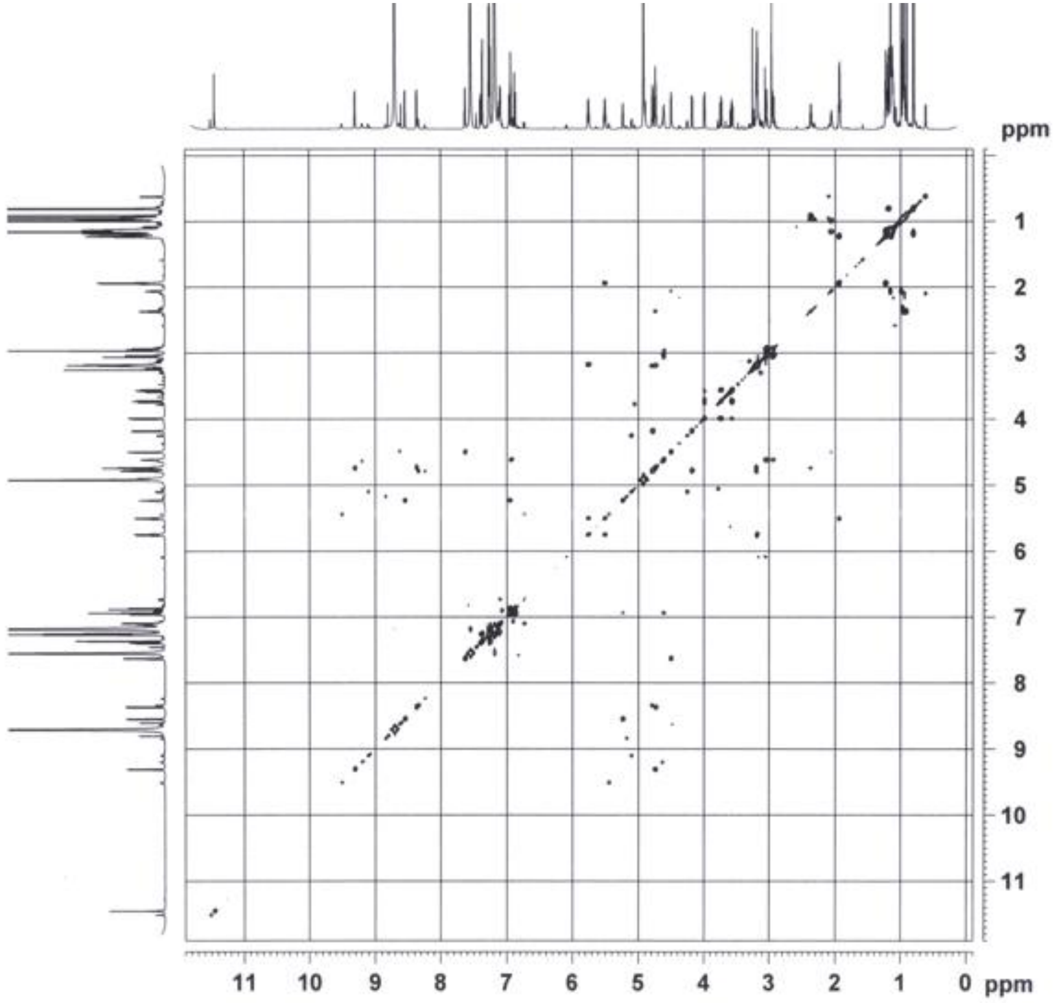

Figure S13. HMQC spectrum (900 MHz) of thalassospiramide D (**3**) in pyridine-d_5.

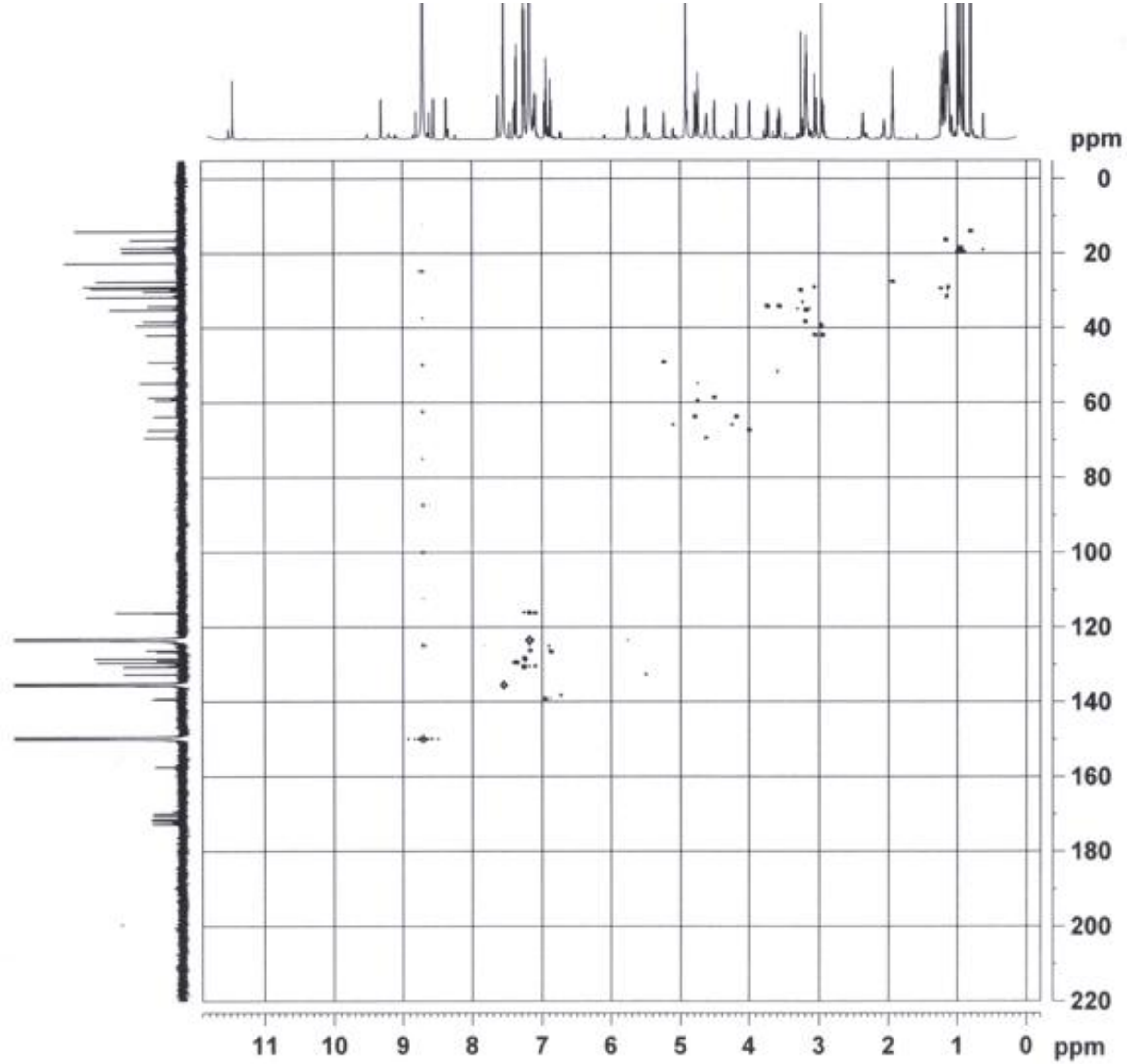

Figure S14. HMBC spectrum (900 MHz) of thalassospiramide D (**3**) in pyridine-d_5.

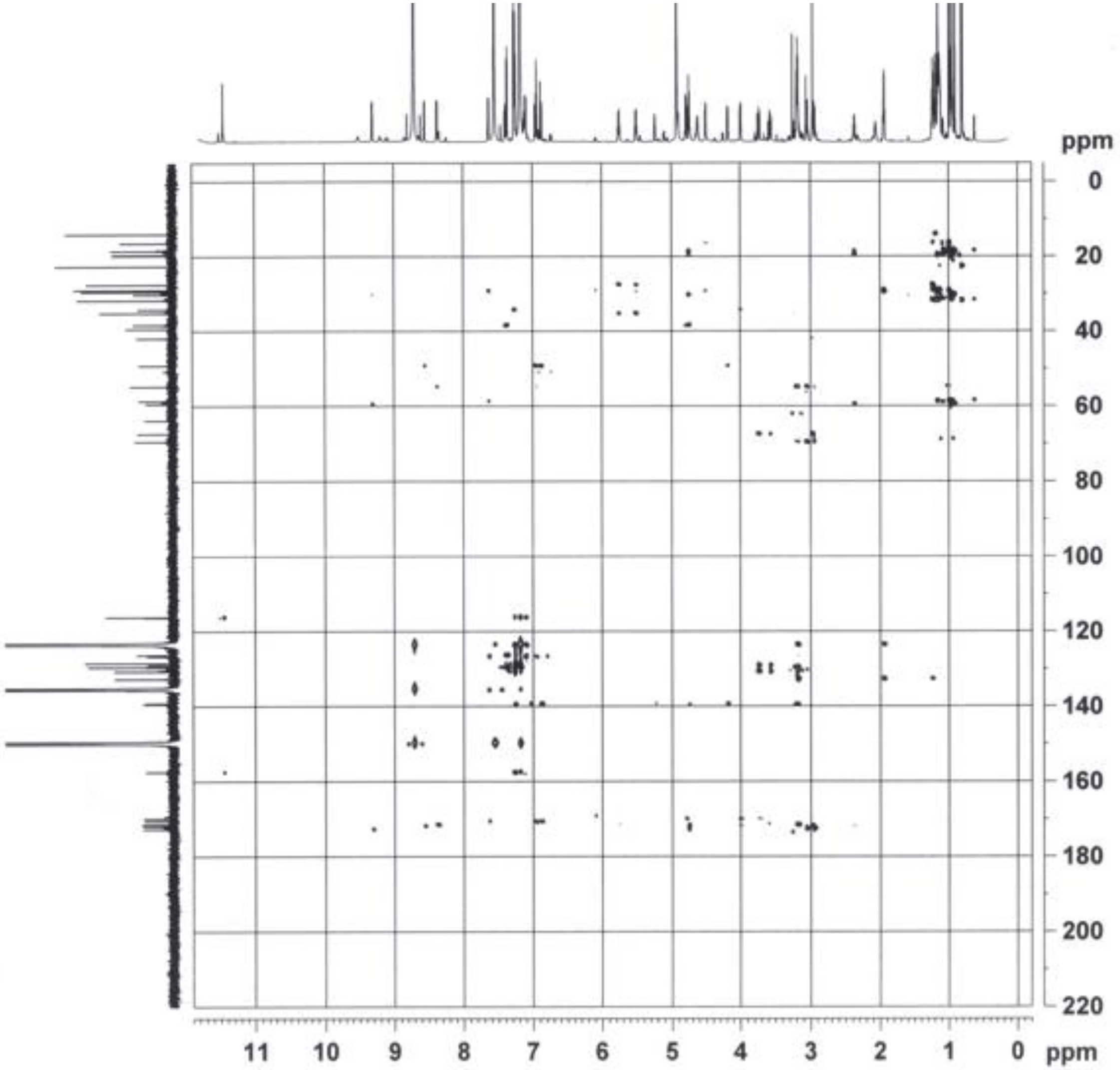

Figure S15. TOCSY spectrum (900 MHz) of thalassospiramide D (**3**) in pyridine-d_5.

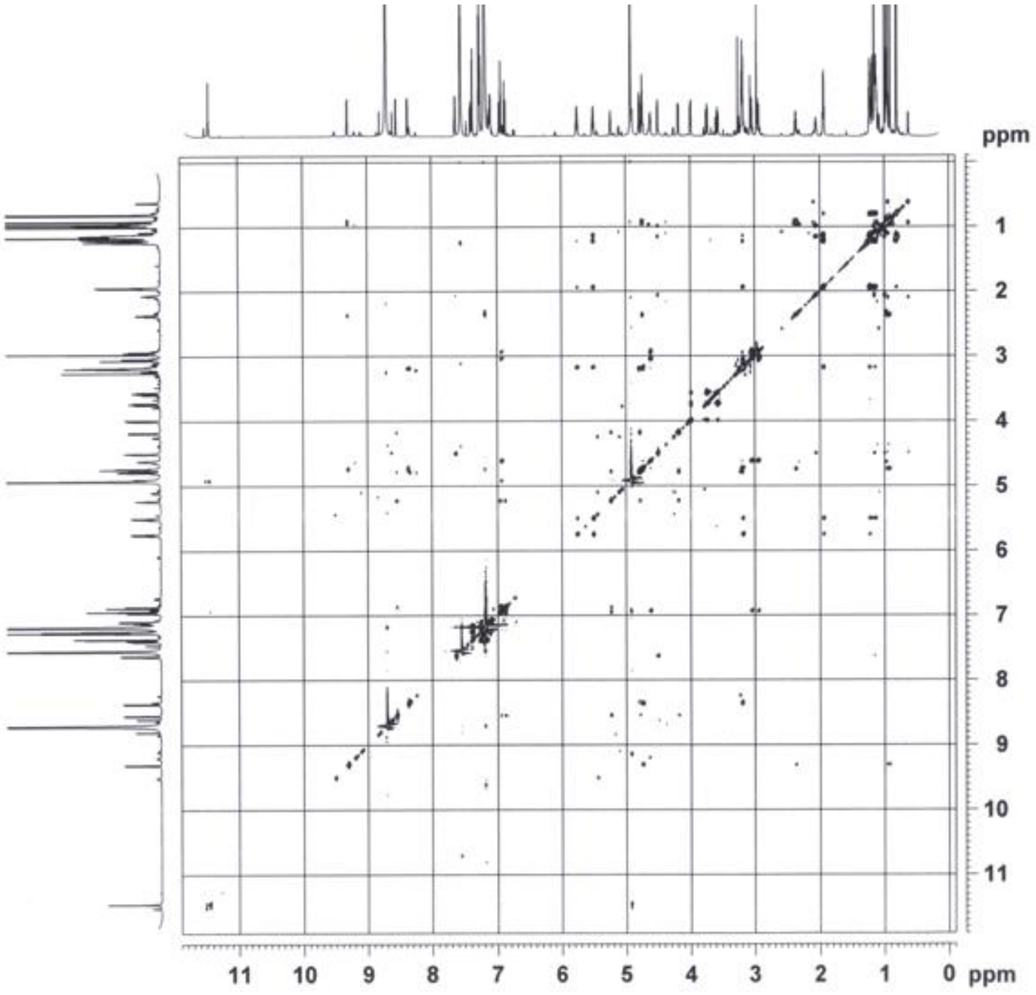

Figure S16. ROESY spectrum (900 MHz) of thalassospiramide D (**3**) in pyridine-d_5.

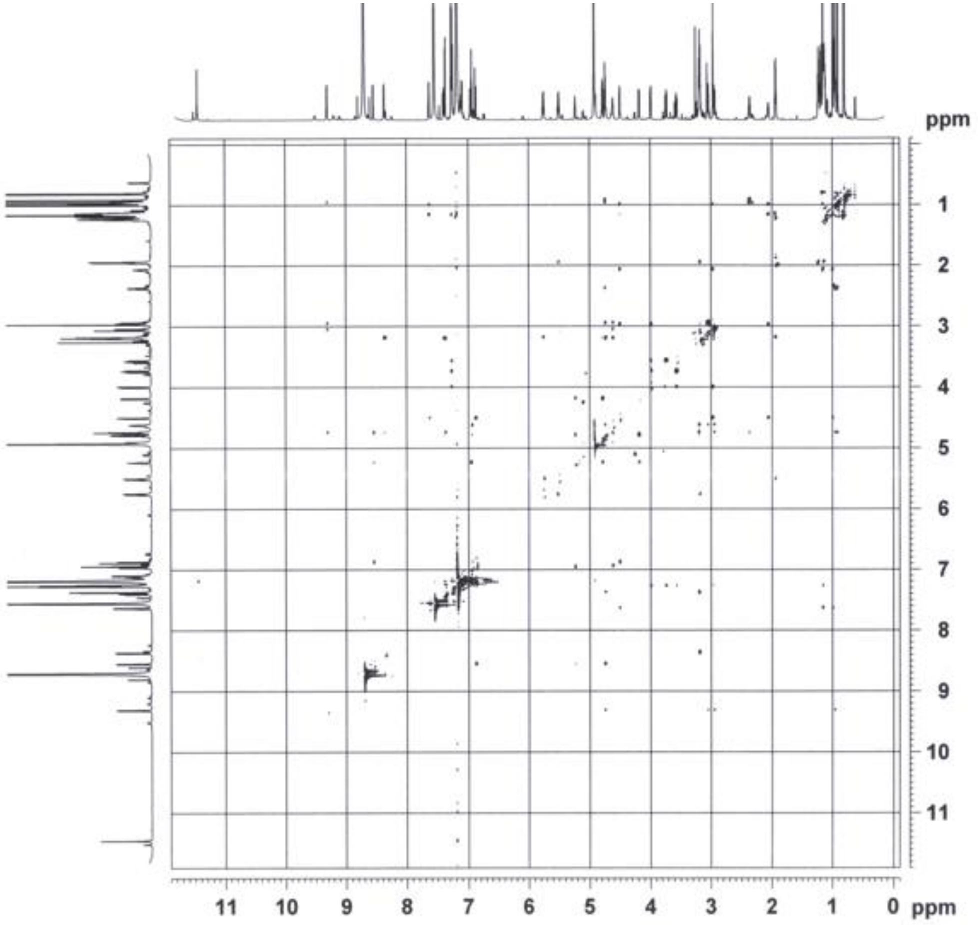

Table S1. NMR Data for **3** in pyridine-d_5.

C/H	δ_H [a]	mult (*J* in Hz)	δ_C [b]	
1			170.1	C
2	4.02	dd (11.0, 4.5)	67.6	CH
3a	3.59	dd (14.0, 4.5)	34.5	CH_2
3b	3.77	dd (14.0, 11.0)		
4			129.2	C
5	7.30	d (8.0)	130.8	CH
6	7.22	m	116.4	CH
7			157.7	C
7-OH	11.40	br. s		
8	7.22	m	116.4	CH
9	7.30	d (8.0)	130.8	CH
10	3.00	s	39.6	CH_3
11			172.0	C
12	4.53	dd (6.0,4.0)	58.9	CH
12-NH	7.66	d (6.0)		
13	2.10	m	29.4	CH
14	1.02	d (7.0)	19.9	CH_3
15	1.18	d (7.0)	16.6	CH_3
16			170.9	C
17	6.91	d (16.0)	126.8	CH
18	6.99	dd (16.0, 5.0)	139.4	CH
19	5.27	br. m	49.4	CH
19-NH	8.57	d (7.5)		
20a	4.82	dd (11.0, 2.0)	64.0	CH_2
20b	4.21	dd (11.0, 2.0)		
21			172.1	C
22	4.77	m	59.7	CH
22-NH	9.34	d (8.0)		
23	2.40	m	30.4	CH
24	0.94	d (7.0)	19.7	CH_3
25	0.98	d (7.0)	18.6	CH_3
26			172.9	C
27a	3.08	dd (14.0, 8.0)	42.1	CH_2
27b	2.96	dd (14.0, 5.0)		
28	4.65	m	69.7	CH
28-OH	6.97	br. s		
29	4.77	m	55.0	CH
29-NH	8.40	d (9.5)		
30	3.22	m	38.5	CH_2
31			139.6	C
32	7.40	d (7.5)	129.8	CH
33	7.29	dd (7.5, 7.5)	128.7	CH
34	7.22	m	126.5	CH
35	7.29	dd (7.5, 7.5)	128.7	CH
36	7.40	d (7.5)	129.8	CH

Table S1. *Cont.*

37			171.7	C
38	3.21	m	35.4	CH_2
39	5.78	dtt (11.0, 7.0, 1.5)	123.4	CH
40	5.53	dtt (11.0, 7.5, 1.5)	132.8	CH
41	1.97	m	27.7	CH_2
42	1.26	m	29.6	CH_2
43	1.16	m	29.2	CH_2
44	1.13	m	31.9	CH_2
45	1.15	m	22.8	CH_2
46	0.84	t (7.0)	14.2	CH_3

[a] 900 MHz; [b] 225 MHz.

Figure S17. Effects of thalassospiramides G, A and D on LPS-induced cell cytotoxicity. RAW264.7 cells were stimulated with 1 μg/mL LPS in the presence or absence of the thalassospiramides for 20 h. Cell cytotoxicity was determined using the MTT method. The data represent the mean ± SD of triplicate experiments.

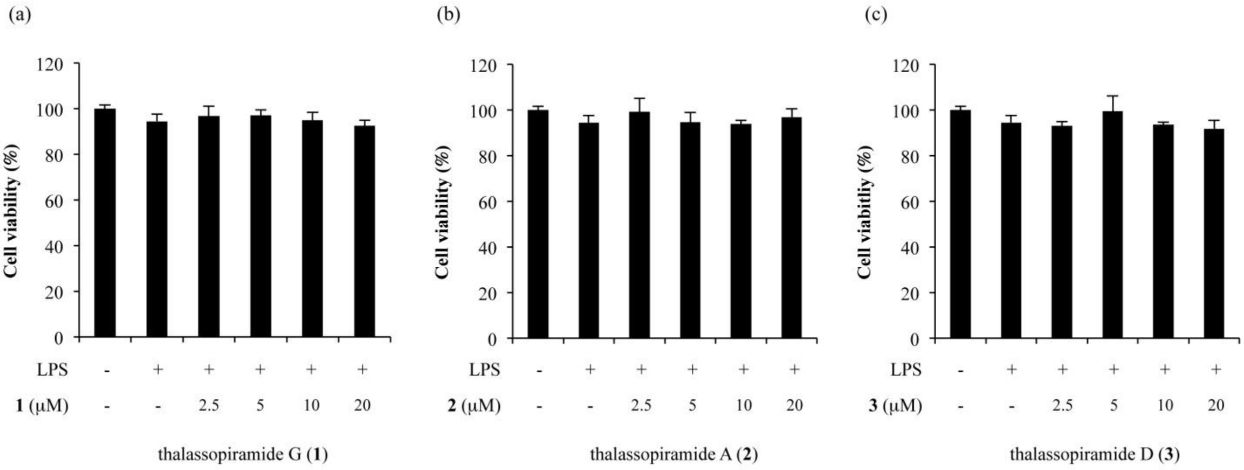

Supplement: Supplementary File 1 — Supplementary Information (PDF, 768 KB) [file marinedrugs-11-00611-s001.pdf]
